# Intensive care doctors and nurses personal preferences for Intensive Care, as compared to the general population: a discrete choice experiment

**DOI:** 10.1186/s13054-021-03712-4

**Published:** 2021-08-10

**Authors:** Matthew H. Anstey, Imogen A. Mitchell, Charlie Corke, Lauren Murray, Marion Mitchell, Andrew Udy, Vineet Sarode, Nhi Nguyen, Oliver Flower, Kwok M. Ho, Edward Litton, Bradley Wibrow, Richard Norman

**Affiliations:** 1 Intensive Care Department, Sir Charles Gairdner Hospital, Level 4 G Block, Hospital Ave, Nedlands, Perth, WA 6009 Australia; 2 School of Population Health, Curtin University, Bentley, Australia; 3 School of Medicine, University of Western Australia, Crawley, Australia; 4 Australian National University, Canberra, Australia; 5 Canberra Hospital, Canberra, Australia; 6 University Hospital Geelong, Geelong, Australia; 7 Sunshine Coast University Hospital, Birtinya, QLD Australia; 8 Griffith University, Griffith, QLD Australia; 9 Princess Alexandra Hospital, Woolloongabba, QLD Australia; 10 Alfred Hospital, Melbourne, Australia; 11 Monash University, Melbourne, Australia; 12 Cabrini Hospital, Melbourne, Australia; 13 Nepean Hospital, Kingswood, NSW Australia; 14 Royal North Shore Hospital, Sydney, Australia; 15 Royal Perth Hospital, Perth, Australia; 16 School of Veterinary and Life Sciences, Murdoch University, Perth, Australia; 17 Fiona Stanley Hospital, Perth, Australia

**Keywords:** Decision making, Discrete choice experiment, Attitude to death, Intensive care units

## Abstract

**Background:**

To test the hypothesis that Intensive Care Unit (ICU) doctors and nurses differ in their personal preferences for treatment from the general population, and whether doctors and nurses make different choices when thinking about themselves, as compared to when they are treating a patient.

**Methods:**

Cross sectional, observational study conducted in 13 ICUs in Australia in 2017 using a discrete choice experiment survey. Respondents completed a series of choice sets, based on hypothetical situations which varied in the severity or likelihood of: death, cognitive impairment, need for prolonged treatment, need for assistance with care or requiring residential care.

**Results:**

A total of 980 ICU staff (233 doctors and 747 nurses) participated in the study. ICU staff place the highest value on avoiding ending up in a dependent state. The ICU staff were more likely to choose to discontinue therapy when the prognosis was worse, compared with the general population. There was consensus between ICU staff personal views and the treatment pathway likely to be followed in 69% of the choices considered by nurses and 70% of those faced by doctors. In 27% (1614/5945 responses) of the nurses and 23% of the doctors (435/1870 responses), they felt that aggressive treatment would be continued for the hypothetical patient but they would not want that for themselves.

**Conclusion:**

The likelihood of returning to independence (or not requiring care assistance) was reported as the most important factor for ICU staff (and the general population) in deciding whether to receive ongoing treatments. Goals of care discussions should focus on this, over likelihood of survival.

**Supplementary Information:**

The online version contains supplementary material available at 10.1186/s13054-021-03712-4.

## Introduction

Shared decision making in the Intensive Care Unit (ICU) for treatment decisions and limitations of care is complicated: most patients are too sick to take part in the decision-making process and many patients have never documented or discussed their wishes with family members [1, 2]. As a result, there are situations where it is difficult to judge the appropriate intensity and duration of treatments to be provided. In making decisions, doctors look to family members to provide their estimate of the patient’s wishes, and they in turn, often ask the doctor, what they think should happen. In the absence of the patient voice, the doctor acts as a guide for the surrogate decision makers [3]. Leaving aside the documented complexities with communication between doctors and surrogate decision makers, understanding the doctor’s personal choices for treatment is important, as well as the ethical climate of the unit in which they work [4, 5]. There is evidence that when faced with a critical illness, some doctors chose less aggressive treatments for themselves, than they would for their patients. Collaborative family meetings, those which involve the direct care nurse, have been shown to improve the quality of communication [4, 6, 7]. This raises the question: do the attitudes of doctors and nurses towards end of life decisions in the intensive care reflect those of patients or their families whom they are guiding? There has not been much prior research on comparing doctors and nurses’ attitudes to those of the general population, on whether their personal preferences could act as substitutes in the absence of documented wishes. The aim of this study was to test the hypothesis that doctors and nurses differ in their own personal preferences for aspects of care from the general population. In addition, we hypothesized that doctors and nurses make different choices when thinking about themselves, as compared to when they are advising about a patient.

## Methods

We conducted a cross sectional study in a voluntary sample of doctors and nurses from 13 participating adult Intensive Care Units across Australia and compared the results to a previously reported survey of the general population using almost identical survey methods [8]. The survey methodology utilized a prioritization technique called a discrete choice experiment (DCE). These experiments offer a flexible and reliable method for quantifying trade-offs that respondents are willing to make [9, 10]. Respondents are asked to complete a series of choice sets in which two or more hypothetical situations are presented, which differ in a number of ways. In this project we aimed to identify the threshold at which people considered certain health states where they would no longer want to continue active treatment, and choose to die [11, 12]. The dimensions used were *loss of functional autonomy*, *likelihood of death, need for prolonged hospital treatment, cognitive disability,* and *degree of burden on others*. Though not exhaustive, these represent areas in which respondents were likely to have strong preferences, (17) and are similar to the Society of Critical Care Medicine policy statement outlining ICU interventions that could be considered inappropriate [13, 14]. Institutional approval was obtained from all participating sites prior to study commencement. The general population data have been reported elsewhere [8]. Briefly, a general population sample of 984 completed an online survey almost identical to the ICU clinician survey (Additional file 2: Supplementary material - Copy of the survey - doctor’s version). Respondents were more likely to state a preference for supportive care (removal of active therapy) if they were likely to die from therapy, if they were likely to have significant ongoing health issues relating to memory/concentration, required considerable care, or would be unable to stay in their own home. The respondent characteristics that predicted a higher willingness to remain on active therapy were being religious and younger.

### Survey development

Additional file 1: Table S1 outlines the survey dimensions, based on common situations that provoke goals of care discussions in the ICU. Recognizing that prognostic information is provided in degrees of likelihood, the likelihoods were defined both in words (low/moderate/high) and numerically (20/50/80% respectively).

**Fig. 1:**
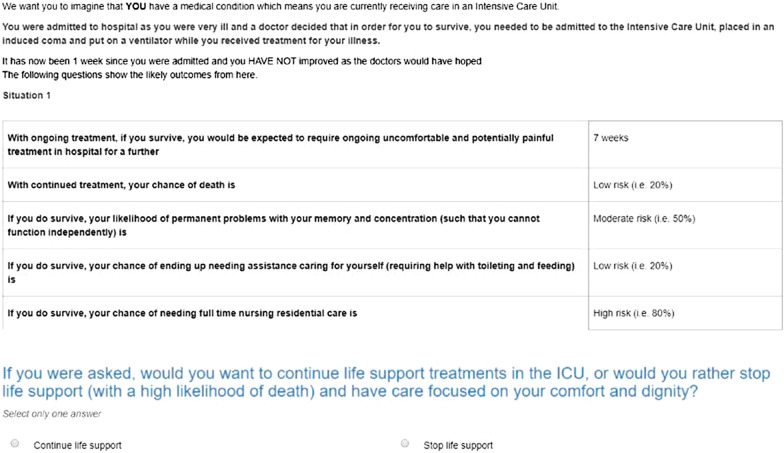
Example of scenario

The ICU survey underwent several rounds of piloting with feedback from a sample of staff from participating sites. An extra section was added to the ICU survey to understand whether perceived unit practice differed from what staff felt they would want for themselves. To understand any discordance between what the respondent would want for themselves, and what might happen with a similar patient (the perceived intensive care unit practices), the ICU survey included the item, “if a patient with this condition was in your intensive care, the medical consensus would favour continuing or talking to the family about stopping active treatment” (Fig. 1). Doctors and nurses were also asked about their personal health, personal experience of ICU, number of dependents, and religious beliefs. Each hospital site participated during a 2-week period between October and December 2017, with voluntary participation from their doctors and nurses. The survey was distributed at each site, and completed online using Survey Engine, a host of DCEs who have conducted a large number of health preference surveys. The ordering of scenarios was randomized in all surveys to reduce response bias. The survey was comparable across the general population and ICU staff to allow comparison between the groups. Several questions were included to assess the respondents understanding and ease with completing the survey.

### Statistical analysis

Descriptive statistics were generated for all variables, where means and standard deviations (SD) are presented for continuous variables and counts and percentages are presented for categorical variables. We tested for differences between doctors and nurses using Chi-Square test (Fisher’s exact test where appropriate) for categorical variables and independent samples t-test for continuous variables. Analysis of the preference data was conducted using a single multivariate logistic regression, which reflects the binary nature of the dependent variable (i.e. the respondent either choses to continue or stop life support). Standard errors were clustered to reflect the fact that respondents completed more than one choice task, meaning that responses were not independent from other responses from the same individual. One level was omitted in each dimension as a reference case, making it implicitly part of the constant term. The best level of each dimension was omitted, so the a priori assumption was that all regression coefficients should be negative. To express this differently, a move from a relatively good level of a dimension to a poorer level (for example a higher chance of dying) should be associated with an increased probability of the respondent opting to stop life support.

To explore the relationship between respondent characteristics and response (e.g. do ICU staff respond differently to the general population), a series of additional analyses was run, looking at respondent characteristics (gender, religious belief, dependents, age, country of birth, primary language, parenthood status). The respondent characteristics were included as a simple constant term, reflecting the association between those characteristics and the willingness of respondents to continue active therapy or not. Each was included as in turn, with those statistically significant at the 5% level included in a larger multivariate analysis. Data were analyzed using STATA 13 (Statacorp 13).

## Results

Characteristics of the ICU sample are outlined in Table 1; 13 hospitals contributed to the study, all metropolitan tertiary teaching hospitals, except for one private hospital and two large regional centers. Of the 233 doctors included in the study, 100 were specialists, 72 were trainee doctors, and 61 were non-trainees. While the demographic questions were optional and some characteristics had missing data, there was no missing data for any of the choice on treatment options. 5.7% (45/785) of staff had previously been admitted to ICU themselves, whereas 41.8% (328/784, 263/598 nurses, 65/186 doctors) had family members previously admitted to ICU.

**Table 1:** Survey respondents (*N* = 980)

Variable	All respondents* (*N* = 980)	Doctors (*n* = 233)	Nurses (*n* = 747)
Female	557 (56.8%)	60 (25.7%)	497 (66.5%)
Born in Australia	521 (53.2%)	81 (34.8%)	440 (58.9%)
Dependents under 18	338 (34.5%)	90 (38.6%)	248 (33.2%)
Somewhat or very religious	267 (27.2%)	54 (23.2%)	213 (28.5%)
Very good or excellent self-reported health	547 (55.8%)	138 (59.2%)	419 (56.1%)

^*^All questions were optional, so may not sum to 980

The DCE results for the ICU groups are presented in Table 2, reporting the odds ratios associated with choosing to decline active therapy in the ICU staff sample (sub-divided into doctors and nurses). A number of important points emerge from these results. In both groups, and in all dimensions, the severity of disability of the patient is positively associated with the likelihood of preferring to discontinue treatment. The odds ratios are highest for discontinuing treatment when the likelihood of ending up in residential care is highest. This is slightly higher for the doctors than the nurses. As the prognosis for each category worsens, the ICU staff are more likely to suggest active therapy should be withdrawn.

**Table 2:** Comparison of odds-ratios for ICU groups choosing to decline active therapy

Dimension	Level	All ICU staff^#^ (*n* = 980)	Doctors (*n* = 233)	Nurses (*n* = 747)
*X*1 (further treatment)	1 week	Reference	Reference	Reference
	4 weeks	1.25 (0.07)*	1.06 (0.11)	1.32 (0.09)*
	7 weeks	1.66 (0.11)*	1.41 (0.20)*	1.74 (0.14)*
*X*2 (chance of death)	Low risk (20%)	Reference	Reference	Reference
	Moderate risk (50%)	1.19 (0.06)*	1.16 (0.11)	1.19 (0.07)*
	High risk (80%)	1.94 (0.13)*	1.65 (0.25)*	2.02 (0.16)*
*X*3 (memory and concentration)	Low risk (20%)	Reference	Reference	Reference
	Moderate risk (50%)	1.58 (0.08)*	1.86 (0.19)*	1.51 (0.09)*
	High risk (80%)	3.76 (0.26)*	4.68 (0.70)*	3.52 (0.27)*
*X*4 (care assistance)	Low risk (20%)	Reference	Reference	Reference
	Moderate risk (50%)	1.63 (0.10)*	1.57 (0.21)*	1.65 (0.11)*
	High risk (80%)	4.02 (0.28)*	4.29 (0.65)*	3.94 (0.32)*
*X*5 (residential care)	Low risk (20%)	Reference	Reference	Reference
	Moderate risk (50%)	1.85 (0.10)*	1.64 (0.18)*	1.92 (0.11)*
	High risk (80%)	6.09 (0.45)*	5.33 (0.75)*	6.41 (0.55)*
Constant		0.12 (0.01)*	0.12 (0.03)*	0.12 (0.02)*
Pseudo *R*^2^		0.142	0.142	0.144

Statistical significance is denoted at the 5% level (*)

Numbers in () represent standard errors

^#^All ICU staff is doctors and nurses

When the respondent characteristics were added to this regression, most did not show a clear pattern (Table 3). In particular, the association with response and age, religion, country of birth, and language was not strong and there was no evidence to reject the null hypothesis that there was no association. Gender and parenthood did predict the choice to continue active treatment. Specifically, male respondents and respondents with children (either younger, defined as under 5, or older, defined as 5–18 years) predicted a preference for continuation of care.

**Table 3:** Addition of select respondent demographics to model predicting choosing to decline active therapy

Dimension	Level	All ICU staff (*n* = 980)
*X*1 (further treatment)	1 week	Reference
	4 weeks	1.28 (0.08)*
	7 weeks	1.70 (0.12)*
*X*2 (chance of death)	Low risk (20%)	Reference
	Moderate risk (50%)	1.20 (0.06)*
	High risk (80%)	2.02 (0.15)*
*X*3 (memory and concentration)	Low risk (20%)	Reference
	Moderate risk (50%)	1.70 (0.09)*
	High risk (80%)	4.03 (0.30)*
*X*4 (care assistance)	Low risk (20%)	Reference
	Moderate risk (50%)	1.66 (0.11)*
	High risk (80%)	4.19 (0.32)*
*X*5 (residential care)	Low risk (20%)	Reference
	Moderate risk (50%)	1.91 (0.11)*
	High risk (80%)	6.54 (0.53)*
Gender	Male	Reference
	Female	1.38 (0.17)*
Children under 5	No	Reference
	Yes	0.58 (0.08)*
Children 15–18	No	Reference
	Yes	0.64 (0.08)*
Constant		0.11 (0.02)*
Pseudo *R*^2^		0.164

Statistical significance is denoted at the 5% level (*)

In looking at the comparison between staff personal views, and the perception of what treatment pathway their intensive care unit would offer, there was general agreement. Nurses felt that the staff consensus would agree with their own personal view in 69% of the responses (4075/5945 responses), and agreement occurred in 70% (1307/1870 responses) of the doctors’ responses. Divergent views were predominantly that the staff felt that aggressive treatment should be continued for the hypothetical patient [nurses 1614/5945 (27%), doctors 435/1870 (23%)], whereas they would not want that for themselves. It was a minority of cases that the ICU staff felt that they would want aggressive treatment but that the unit would pursue a palliative approach [nurses 256/5945 (4%), doctors 128/1870 (6.8%)]. Additional file 1: Table S2 outlines the odds ratios for these different options.

Respondents comprehension of the survey was assessed using a 5-point Likert scale, with 65% (499/793) finding the description and questions about the scenarios as either clear or very clear (5% very unclear). Answering the tradeoff questions was more difficult for respondents, with 52% (415/793) finding them difficult or very difficult.

## Discussion

In this study of 980 doctors and nurses surveyed in 13 centres, the likelihood of returning to independence (or not requiring care assistance) was reported as the most important factor in deciding whether they would want to receive ongoing treatments in the ICU.

One of the key discussion points for this study is the ability to compare the results of the ICU staff to that of a general population sample, who undertook almost an identical survey using the same methodology. The respondents were similarly matched except the general population was more likely to have personally been previously admitted to ICU (17.9% vs 5.7%) but both samples were equally likely to have known someone personally admitted to ICU (42.5% vs 41.8%). In all groups, and in all dimensions, the severity of disability of the patient is positively associated with the likelihood of preferring to discontinue treatment. However, the results between the populations differ in some important regards. First, the constant is higher in the general population. This means that, if the two samples consider a person at the reference level in each dimension (which equates to the best prognosis), then the general population is more likely to choose to discontinue therapy. Conversely, the odds ratios are higher in both ICU staff samples; this means that as prognosis worsens the impact on choice is higher in the ICU staff sample than the general population (for example, for the highest risk category for ending up requiring residential care, the odds ratio for medical staff 6.088, general population 2.07). Indeed, for someone with a relatively poor prognosis, the ICU staff are more likely to suggest active therapy should be withdrawn. Conversely, the ICU staff were less likely to prefer discontinuing treatment than the general population when considering patients with relatively good prognosis, as shown by the difference in the constant. Thus, the ICU staff sample’s responses are more driven by the specific prognosis of the individual; this is reflected in the higher Pseudo R^2^ value relative to that seen in the general population.

There was perhaps less difference between the attitudes of doctors and nurses than we might have expected [6, 15]. The need for further prolonged treatment was a more important factor in the nurse group than in the doctor group.

In the majority of cases, there was consensus between the preference of what the ICU staff member would want for themselves, and their perception of what would actually happen in their ICU. This contrasts to work from the United States that showed a larger disconnect between what clinicians would want for themselves and what would actually happen [16]. This may reflect differences between countries in the availability of ICU beds, admission policies and the practice of end of life care [17]. Furthermore, it may also be that differences in the life experiences (such as prior exposure to Intensive Care, trust in the healthcare system, insurance status or disability) of clinicians and the general public may lead to different results in other countries. While Australian clinicians appear to be reasonable proxies of patient preferences, they remain imperfect, as 30% discordance was reported between the staff and general population answers. Within this group, 25% of staff preferred less aggressive treatments for themselves that what they expected would happen in their unit, which is a sizeable number.

Our results suggest that being a parent makes ICU staff more inclined to continue treatment. Previous Canadian research showed that individual characteristics of clinicians (years of experience), and units (size) were as predictive of likelihood to withdraw care as patient characteristics, with no differences between nursing and medical staff [18]. This supports the idea that is can be both an individual’s values, but also the setting and culture within which they work, that can influence their decision making. Despite increased attention to advance care planning, current research still suggests that the likelihood a patient is admitted to ICU remains variable, as is eliciting family preferences and values in regards to care decisions [19–22]. It may be that continuation of active treatment in the ICU is also subject to similar factors. The results of this survey are useful in reinforcing the need for developing unit level strategies that support family involvement in end of life decision-making, and initiatives to overcome any real or perceived barriers to goals of care discussions [23, 24]. The results of this study highlight the need for reporting outcomes beyond mortality including the likelihood of significant ongoing disability, as it is these outcomes that really seem to matter to patients [25, 26].

There are several limitations. First, the general population and the ICU staff come from an Australian sample, and thus the results may not be generalizable to other countries. Second, the use of hypothetical scenarios may not entirely reflect what would happen in real life, but they were developed in conjunction with ICU staff after an iterative process, and were the same as those used with the general population. The study method also provided the opportunity for a large number of respondents. Third, the use of preference surveys may be potentially limited by the difficulty in comparing the different trade-offs. Finally, we did not explicitly ask about experience with disability, which may alter a respondent’s willingness to accept an outcome that includes a disability.

## Conclusions

Similar to the general population, the most important outcome variable influencing ICU staff is the likelihood to return to independence following critical illness. While what ICU staff would want for themselves aligns with what they believe would apply for a majority of patients, there remains a sizeable number of scenarios where ICU staff would prefer less aggressive treatments if they were the patients themselves. This result reinforces the need for an increased use of advanced care directives or, at least, encouraging individuals to discuss their preferences in goals of care when they are critically ill—not just for patients but the healthcare providers as well. Only then, early meaningful family involvement in setting patient-centered goals of care for ICU patients can be fully materialized.

## Supplementary Information

**Additional file 1**. Supplementary Table 1: Dimensions and levels in the experiment.

**Additional file 2**. Supplementary material - Copy of the survey - doctor’s version

## Data Availability

The datasets generated and/or analyzed during the current study are not publicly available due to ethics approvals but are available from the corresponding author on reasonable request.
