# Supplementary material for: Intensive care doctors and nurses personal preferences for Intensive Care, as compared to the general population: a discrete choice experiment
Source: Crit Care. 2021 Aug 10;25:287. doi: 10.1186/s13054-021-03712-4 (PMC8353726; doi:10.1186/s13054-021-03712-4)
Supplement: Supplementary file 1 — Additional file 1. Supplementary Table 1: Dimensions and levels in the experiment. [file 13054_2021_3712_MOESM1_ESM.docx]

**Supplementary Table 1: Dimensions and levels in the experiment**

| Dimension | Best level (0) | Intermediate level (1) | Worst level (2) |
| --- | --- | --- | --- |
| X1 With ongoing treatment, if you survive, you would be expected to require ongoing uncomfortable and potentially painful treatment in hospital for a further | 1 week | 4 weeks | 7 weeks |
| X2 With continued treatment, your chance of death is | Low risk (i.e. 20%) | Moderate risk (i.e. 50%) | High risk (i.e. 80%) |
| X3 If you do survive, your likelihood of permanent problems with your memory and concentration (such that you cannot function independently) is | Low risk (i.e. 20%) | Moderate risk (i.e. 50%) | High risk (i.e. 80%) |
| X4 If you do survive, your chance of ending up needing assistance caring for yourself (requiring help with toileting and feeding) is | Low risk (i.e. 20%) | Moderate risk (i.e. 50%) | High risk (i.e. 80%) |
| X5 If you do survive, your chance of needing full time nursing residential care is | Low risk (i.e. 20%) | Moderate risk (i.e. 50%) | High risk (i.e. 80%) |

**Supplementary Table 2. Perceived likelihood of patient to receive ongoing treatment in the ICU.**

| Dimension | Level | Doctors | Nurses |
| --- | --- | --- | --- |
| X1 (Further treatment) | 1 week |  |  |
|  | 4 weeks | 1.165 (0.146) | 1.374 (0.088)* |
|  | 7 weeks | 1.409 (0.197)* | 1.696 (0.130)* |
| X2 (Chance of death) | Low risk (20%) |  |  |
|  | Moderate risk (50%) | 1.41 (0.146)* | 1.28 (0.072)* |
|  | High risk (80%) | 2.73 (0.40)* | 2.796 (0.211)* |
| X3 (Memory and concentration) | Low risk (20%) |  |  |
|  | Moderate risk (50%) | 1.69 (0.189)* | 1.530 (0.094)* |
|  | High risk (80%) | 3.14 (0.407)* | 2401 (0.159)* |
| X4 (Care assistance) | Low risk (20%) |  |  |
|  | Moderate risk (50%) | 1.41 (0.193)* | 1.23 (0.095)* |
|  | High risk (80%) | 3.08 (0.446)* | 2.39 (0.182)* |
| X5 (Residential care) | Low risk (20%) |  |  |
|  | Moderate risk (50%) | 1.43 (0.164)* | 1.52(0.099)* |
|  | High risk (80%) | 4.59 (0.589)* | 3.83 (0.268)* |
| Constant |  | 0.006 (0.015)* | 0.065 (0.009)* |

Statistical significance is denoted at the 5% level (*)
