# Supplementary material for: Intensive care doctors and nurses personal preferences for Intensive Care, as compared to the general population: a discrete choice experiment
Source: Crit Care. 2021 Aug 10;25:287. doi: 10.1186/s13054-021-03712-4 (PMC8353726; doi:10.1186/s13054-021-03712-4)
Supplement: Supplementary file 2 — Additional file 2. Supplementary material - Copy of the survey - doctor’s version [file 13054_2021_3712_MOESM2_ESM.pdf]

Patient and population preferences for intensive care: Integrating the person into decisions around care

Welcome

We are inviting you to participate in a study about how people value health in the context of care within an Intensive Care Unit (ICU). We are exploring whether the way doctors and nurses feel about the health of people in ICU is the same as the rest of the population. **The survey is going to ask you to imagine that you are critically ill and what your wishes would be in a variety of different hypothetical outcomes. Your responses to these hypothetical questions will be used to help us understand what is most important to people when considering their health.** This study is being undertaken by researchers based at Sir Charles Gairdner Hospital in Perth, and Curtin University. If you would like to speak to someone about the study or this survey, please call Dr Richard Norman on (08) 92661327. If the questions in the survey make you feel anxious or depressed, we recommend you consult your GP or call Beyond Blue on 1300 22 4636 or Lifeline 13 11 14.

The survey will take about 15 minutes. Your participation in this study is entirely voluntary. By completing the survey you are voluntarily agreeing to participate. You can also choose to withdraw from the study at any time without any consequences. The survey software has been designed to protect your privacy and is secure. The survey is anonymous and confidential. No one will be able to identify you or your answers, and no one will know whether or not you participated in the study. Your survey responses will be stored for the purposes of analysis, and combined with those from other participants and analyzed as a group.

Please enter your site code

prev

next

© 2018 SurveyEngine P/L. Version 3.1

Doctor version of the study

2. Role I

What is your role in the ICU?

Select only one answer

- Nurse
- Doctor

What is your medical specialty?

Select only one answer

- Intensive care specialist
- ICU trainee
- Career medical officer
- Non-ICU trainee (resident or registrar)

Page 4 consists of background survey logic and is not shown to respondents

We want you to imagine that **YOU** have a medical condition which means you are currently receiving care in an Intensive Care Unit.

You were admitted to hospital as you were very ill and a doctor decided that in order for you to survive, you needed to be admitted to the Intensive Care Unit, placed in an induced coma and put on a ventilator while you received treatment for your illness.

It has now been 1 week since you were admitted and you HAVE NOT improved as the doctors would have hoped.
The following questions show the likely outcomes from here.

Situation 1

**With ongoing treatment, if you survive, you would be expected to require ongoing uncomfortable and potentially painful treatment in hospital for a further**	7 weeks
**With continued treatment, your chance of death is**	Low risk (i.e. 20%)
**If you do survive, your likelihood of permanent problems with your memory and concentration (such that you cannot function independently) is**	Moderate risk (i.e. 50%)
**If you do survive, your chance of ending up needing assistance caring for yourself (requiring help with toileting and feeding) is**	Low risk (i.e. 20%)
**If you do survive, your chance of needing full time nursing residential care is**	High risk (i.e. 80%)

If you were asked, would you want to continue life support treatments in the ICU, or would you rather stop life support (with a high likelihood of death) and have care focused on your comfort and dignity?

Select only one answer

○ Continue life support

○ Stop life support

If a PATIENT with this condition was in your intensive care unit, the medical consensus would favour…

Select only one answer

- ○ Continuing to actively treat the patient
- ○ Talking to the family about stopping active treatment

Page 7 consists of background survey logic and is not shown to respondents

We want you to imagine that **YOU** have a medical condition which means you are currently receiving care in an Intensive Care Unit.

You were admitted to hospital as you were very ill and a doctor decided that in order for you to survive, you needed to be admitted to the Intensive Care Unit, placed in an induced coma and put on a ventilator while you received treatment for your illness.

It has now been 1 week since you were admitted and you HAVE NOT improved as the doctors would have hoped.
The following questions show the likely outcomes from here.

Situation 2

**With ongoing treatment, if you survive, you would be expected to require ongoing uncomfortable and potentially painful treatment in hospital for a further**	7 weeks
**With continued treatment, your chance of death is**	Moderate risk (i.e. 50%)
**If you do survive, your likelihood of permanent problems with your memory and concentration (such that you cannot function independently) is**	Moderate risk (i.e. 50%)
**If you do survive, your chance of ending up needing assistance caring for yourself (requiring help with toileting and feeding) is**	High risk (i.e. 80%)
**If you do survive, your chance of needing full time nursing residential care is**	Low risk (i.e. 20%)

If you were asked, would you want to continue life support treatments in the ICU, or would you rather stop life support (with a high likelihood of death) and have care focused on your comfort and dignity?

Select only one answer

- Continue life support
- Stop life support

If a PATIENT with this condition was in your intensive care unit, the medical consensus would favour…

Select only one answer

- Continuing to actively treat the patient
- Talking to the family about stopping active treatment

Page 10 consists of background survey logic and is not shown to respondents

We want you to imagine that **YOU** have a medical condition which means you are currently receiving care in an Intensive Care Unit.

You were admitted to hospital as you were very ill and a doctor decided that in order for you to survive, you needed to be admitted to the Intensive Care Unit, placed in an induced coma and put on a ventilator while you received treatment for your illness.

It has now been 1 week since you were admitted and you HAVE NOT improved as the doctors would have hoped.
The following questions show the likely outcomes from here.

Situation 3

**With ongoing treatment, if you survive, you would be expected to require ongoing uncomfortable and potentially painful treatment in hospital for a further**	1 week
**With continued treatment, your chance of death is**	High risk (i.e. 80%)
**If you do survive, your likelihood of permanent problems with your memory and concentration (such that you cannot function independently) is**	High risk (i.e. 80%)
**If you do survive, your chance of ending up needing assistance caring for yourself (requiring help with toileting and feeding) is**	Low risk (i.e. 20%)
**If you do survive, your chance of needing full time nursing residential care is**	Moderate risk (i.e. 50%)

If you were asked, would you want to continue life support treatments in the ICU, or would you rather stop life support (with a high likelihood of death) and have care focused on your comfort and dignity?

Select only one answer

- Continue life support
- Stop life support

If a PATIENT with this condition was in your intensive care unit, the medical consensus would favour…

Select only one answer

- Continuing to actively treat the patient
- Talking to the family about stopping active treatment

Page 13 consists of background survey logic and is not shown to respondents

41%

We want you to imagine that **YOU** have a medical condition which means you are currently receiving care in an Intensive Care Unit.

You were admitted to hospital as you were very ill and a doctor decided that in order for you to survive, you needed to be admitted to the Intensive Care Unit, placed in an induced coma and put on a ventilator while you received treatment for your illness.

It has now been 1 week since you were admitted and you HAVE NOT improved as the doctors would have hoped.
The following questions show the likely outcomes from here.

Situation 4

**With ongoing treatment, if you survive, you would be expected to require ongoing uncomfortable and potentially painful treatment in hospital for a further**	1 week
**With continued treatment, your chance of death is**	Low risk (i.e. 20%)
**If you do survive, your likelihood of permanent problems with your memory and concentration (such that you cannot function independently) is**	Moderate risk (i.e. 50%)
**If you do survive, your chance of ending up needing assistance caring for yourself (requiring help with toileting and feeding) is**	High risk (i.e. 80%)
**If you do survive, your chance of needing full time nursing residential care is**	Moderate risk (i.e. 50%)

If you were asked, would you want to continue life support treatments in the ICU, or would you rather stop life support (with a high likelihood of death) and have care focused on your comfort and dignity?

Select only one answer

- ○ Continue life support
- ○ Stop life support

If a PATIENT with this condition was in your intensive care unit, the medical consensus would favour…

Select only one answer

- Continuing to actively treat the patient
- Talking to the family about stopping active treatment

Page 16 consists of background survey logic and is not shown to respondents

We want you to imagine that **YOU** have a medical condition which means you are currently receiving care in an Intensive Care Unit.

You were admitted to hospital as you were very ill and a doctor decided that in order for you to survive, you needed to be admitted to the Intensive Care Unit, placed in an induced coma and put on a ventilator while you received treatment for your illness.

It has now been 1 week since you were admitted and you HAVE NOT improved as the doctors would have hoped.
The following questions show the likely outcomes from here.

Situation 5

**With ongoing treatment, if you survive, you would be expected to require ongoing uncomfortable and potentially painful treatment in hospital for a further**	4 weeks
**With continued treatment, your chance of death is**	High risk (i.e. 80%)
**If you do survive, your likelihood of permanent problems with your memory and concentration (such that you cannot function independently) is**	Moderate risk (i.e. 50%)
**If you do survive, your chance of ending up needing assistance caring for yourself (requiring help with toileting and feeding) is**	Low risk (i.e. 20%)
**If you do survive, your chance of needing full time nursing residential care is**	Low risk (i.e. 20%)

If you were asked, would you want to continue life support treatments in the ICU, or would you rather stop life support (with a high likelihood of death) and have care focused on your comfort and dignity?

Select only one answer

- ○ Continue life support
- ○ Stop life support

If a PATIENT with this condition was in your intensive care unit, the medical consensus would favour…

Select only one answer

- Continuing to actively treat the patient
- Talking to the family about stopping active treatment

Page 19 consists of background survey logic and is not shown to respondents

58%

We want you to imagine that **YOU** have a medical condition which means you are currently receiving care in an Intensive Care Unit.

You were admitted to hospital as you were very ill and a doctor decided that in order for you to survive, you needed to be admitted to the Intensive Care Unit, placed in an induced coma and put on a ventilator while you received treatment for your illness.

It has now been 1 week since you were admitted and you HAVE NOT improved as the doctors would have hoped.
The following questions show the likely outcomes from here.

Situation 6

**With ongoing treatment, if you survive, you would be expected to require ongoing uncomfortable and potentially painful treatment in hospital for a further**	4 weeks
**With continued treatment, your chance of death is**	Low risk (i.e. 20%)
**If you do survive, your likelihood of permanent problems with your memory and concentration (such that you cannot function independently) is**	Low risk (i.e. 20%)
**If you do survive, your chance of ending up needing assistance caring for yourself (requiring help with toileting and feeding) is**	Moderate risk (i.e. 50%)
**If you do survive, your chance of needing full time nursing residential care is**	High risk (i.e. 80%)

If you were asked, would you want to continue life support treatments in the ICU, or would you rather stop life support (with a high likelihood of death) and have care focused on your comfort and dignity?

Select only one answer

- Continue life support
- Stop life support

If a PATIENT with this condition was in your intensive care unit, the medical consensus would favour…

Select only one answer

- Continuing to actively treat the patient
- Talking to the family about stopping active treatment

Page 22 consists of background survey logic and is not shown to respondents

67%

We want you to imagine that **YOU** have a medical condition which means you are currently receiving care in an Intensive Care Unit.

You were admitted to hospital as you were very ill and a doctor decided that in order for you to survive, you needed to be admitted to the Intensive Care Unit, placed in an induced coma and put on a ventilator while you received treatment for your illness.

It has now been 1 week since you were admitted and you HAVE NOT improved as the doctors would have hoped.
The following questions show the likely outcomes from here.

Situation 7

**With ongoing treatment, if you survive, you would be expected to require ongoing uncomfortable and potentially painful treatment in hospital for a further**	4 weeks
**With continued treatment, your chance of death is**	Moderate risk (i.e. 50%)
**If you do survive, your likelihood of permanent problems with your memory and concentration (such that you cannot function independently) is**	High risk (i.e. 80%)
**If you do survive, your chance of ending up needing assistance caring for yourself (requiring help with toileting and feeding) is**	Low risk (i.e. 20%)
**If you do survive, your chance of needing full time nursing residential care is**	High risk (i.e. 80%)

If you were asked, would you want to continue life support treatments in the ICU, or would you rather stop life support (with a high likelihood of death) and have care focused on your comfort and dignity?

Select only one answer

- Continue life support
- Stop life support

If a PATIENT with this condition was in your intensive care unit, the medical consensus would favour…

Select only one answer

- Continuing to actively treat the patient
- Talking to the family about stopping active treatment

Page 25 consists of background survey logic and is not shown to respondents

We want you to imagine that **YOU** have a medical condition which means you are currently receiving care in an Intensive Care Unit.

You were admitted to hospital as you were very ill and a doctor decided that in order for you to survive, you needed to be admitted to the Intensive Care Unit, placed in an induced coma and put on a ventilator while you received treatment for your illness.

It has now been 1 week since you were admitted and you HAVE NOT improved as the doctors would have hoped.
The following questions show the likely outcomes from here.

Situation 8

**With ongoing treatment, if you survive, you would be expected to require ongoing uncomfortable and potentially painful treatment in hospital for a further**	1 week
**With continued treatment, your chance of death is**	Moderate risk (i.e. 50%)
**If you do survive, your likelihood of permanent problems with your memory and concentration (such that you cannot function independently) is**	Low risk (i.e. 20%)
**If you do survive, your chance of ending up needing assistance caring for yourself (requiring help with toileting and feeding) is**	High risk (i.e. 80%)
**If you do survive, your chance of needing full time nursing residential care is**	High risk (i.e. 80%)

If you were asked, would you want to continue life support treatments in the ICU, or would you rather stop life support (with a high likelihood of death) and have care focused on your comfort and dignity?

Select only one answer

- ○ Continue life support
- ○ Stop life support

If a PATIENT with this condition was in your intensive care unit, the medical consensus would favour…

Select only one answer

- Continuing to actively treat the patient
- Talking to the family about stopping active treatment

We want you to imagine that **YOU** have a medical condition which means you are currently receiving care in an Intensive Care Unit.

You were admitted to hospital as you were very ill and a doctor decided that in order for you to survive, you needed to be admitted to the Intensive Care Unit, placed in an induced coma and put on a ventilator while you received treatment for your illness.

It has now been 1 week since you were admitted and you HAVE NOT improved as the doctors would have hoped.
The following questions show the likely outcomes from here.

Situation 9

**With ongoing treatment, if you survive, you would be expected to require ongoing uncomfortable and potentially painful treatment in hospital for a further**	1 week
**With continued treatment, your chance of death is**	Moderate risk (i.e. 50%)
**If you do survive, your likelihood of permanent problems with your memory and concentration (such that you cannot function independently) is**	High risk (i.e. 80%)
**If you do survive, your chance of ending up needing assistance caring for yourself (requiring help with toileting and feeding) is**	Moderate risk (i.e. 50%)
**If you do survive, your chance of needing full time nursing residential care is**	Low risk (i.e. 20%)

If you were asked, would you want to continue life support treatments in the ICU, or would you rather stop life support (with a high likelihood of death) and have care focused on your comfort and dignity?

Select only one answer

- Continue life support
- Stop life support

If a PATIENT with this condition was in your intensive care unit, the medical consensus would favour…

Select only one answer

- Continuing to actively treat the patient
- Talking to the family about stopping active treatment

You have now completed the 9 questions. Thank you.

How clear was the presentation of the health states?

Select only one answer

Very unclear	Unclear	Neither clear nor unclear	Clear	Very clear
○	○	○	○	○

How difficult was it to choose whether to receive the intervention or not?

Select only one answer

Very difficult	Difficult	Neither easy nor difficult	Easy	Very easy
○	○	○	○	○

Section C: Information about you

You have now completed the main part of the experiment. Thank you.
Before we finish, we need you to answer a few more questions about you. This is to ensure we have a good spread of respondents.

What is your sex?

Select only one answer

- male
- female

What is your current age (in years)?

What is your country of birth?

Select only one answer

- Australia
- Other English-speaking country
- Other NON-English-speaking country

What is your main language?

Select only one answer

- ○ English
- ○ Other [ ]

Which of these describe your current living arrangements? Tick all that apply

Select all that apply

- ☐ I live alone
- ☐ I live with my spouse/partner
- ☐ I live with my children
- ☐ I live with my parent(s)
- ☐ I live with my sibling(s)
- ☐ I live with other adult family members
- ☐ I live with other adults (not family members)

How many children do you have in the following age ranges?

Select one response from each row

	No children in this age range	1 child	2 children	3 or more children
Less than five years old	○	○	○	○
Five to eighteeen years old	○	○	○	○

Would you describe yourself as

Select only one answer

- ○ Very religious
- ○ Somewhat religious
- ○ Not religious
- ○ Very non religious

As far as you know, do you have any of the following health conditions at the present time?

Select all that apply

- ☐ Asthma, emphysema, or chronic bronchitis
- ☐ Arthritis or rheumatism
- ☐ Cancer diagnosed in last 3 years
- ☐ Diabetes
- ☐ Digestive problems (such as ulcer, colitis, or gallbladder disease)
- ☐ Heart trouble (such as angina, congestive heart failure, or coronary artery disease)
- ☐ HIV illness or AIDS
- ☐ Kidney disease
- ☐ Liver problems (such as cirrhosis)
- ☐ Stroke
- ☐ None of these

Have you ever been admitted to an Intensive Care Unit?

Select only one answer

- Yes
- No

Has a member of your immediate family ever been admitted to an Intensive Care Unit?

Select only one answer

- Yes
- No

prev

next

© 2018 SurveyEngine P/L. Version 3.1

PAIN / DISCOMFORT: Please select the one option that best describes your health TODAY

Select only one answer

- ◯ I have no pain or discomfort
- ◯ I have slight pain or discomfort
- ◯ I have moderate pain or discomfort
- ◯ I have severe pain or discomfort
- ◯ I have extreme pain or discomfort

ANXIETY / DEPRESSION: Please select the one option that best describes your health TODAY

Select only one answer

- ◯ I am not anxious or depressed
- ◯ I am slightly anxious or depressed
- ◯ I am moderately anxious or depressed
- ◯ I am severely anxious or depressed
- ◯ I am extremely anxious or depressed

In general would you say that your health is:

Select only one answer

Excellent	Very good	Good	Fair	Poor
○	○	○	○	○

Do you agree or disagree with the following statements?

Select one response from each row

	Strongly agree	Agree	Neither agree nor disagree	Disagree	Strongly disagree	Don't know/refuse
I am asked to do an excessive amount of work.	○	○	○	○	○	○
I have thoughts about leaving my current position/job.	○	○	○	○	○	○
In my ICU, death is perceived as a treatment failure, so decisions to withdraw or withhold therapy are seldom made.	○	○	○	○	○	○
In general, I think that the ICU is the best place to provide a good death.	○	○	○	○	○	○

prev next

© 2018 SurveyEngine P/L. Version 3.1

Thank you

You have now finished the task. We appreciate your time and your contribution. To help us improve this survey, we would value your thoughts about how you answered this survey, any aspects you found difficult, or any other thoughts.

If you have any specific comments relating to the survey, we would really valuable your input in the box below.

Enter text below

prev

submit answers and finish

© 2018 SurveyEngine P/L. Version 3.1

Thank You

That concludes the survey

prev

submit answers and finish

© 2018 SurveyEngine P/L. Version 3.1
